# Supplementary material for: Sensorless model-based tension control for a cable-driven exosuit
Source: Wearable Technol. 2024 Dec 10;5:e22. doi: 10.1017/wtc.2024.21 (PMC11729527; doi:10.1017/wtc.2024.21)
Supplement: Bardi et al. supplementary material [file S2631717624000215sup001.pdf]

Sensorless model-based tension control for a cable-driven exosuit

Supplementary materials

1 Transmission model

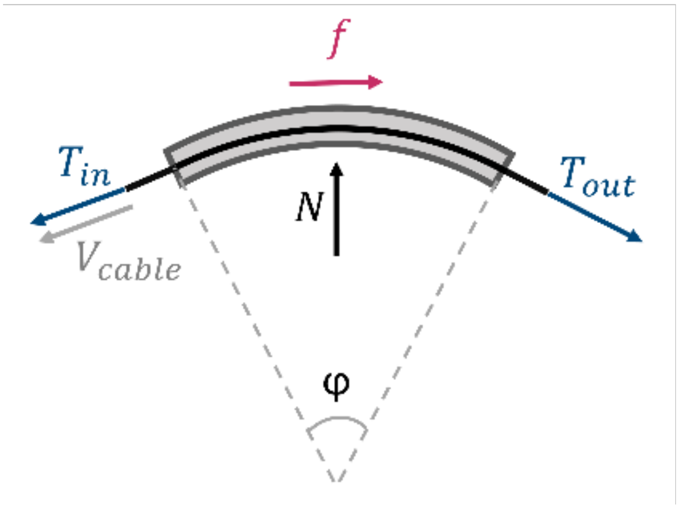

Figure S1: Free-body diagram of the exosuit cable inside the Bowden sheath. T_{in} is the input tension on the TDU side, T_{out} is the output tension on the limb side, N is the normal reaction force acting on the cable, f is the friction force, and ϕ is the total wrap angle of the sheath.

The free-body diagram of the Bowden-sheath cable system is shown in Figure S1. The normal reaction force N acting on the cable from the interaction with the sheath can be approximated as:

$$N = (T_{in} + T_{out}) sin\left(\frac{\phi}{2}\right) \tag{1}$$

where T_{in} is the input tendon tension on the motor side, T_{out} is the output tendon tension acting on the limb, ϕ is the total wrap angle of the sheath. From the equilibrium of the forces acting on the cable, it is possible to express the cable output tension as:

$$T_{out} = T_{in} - f \tag{2}$$

where f is the friction force acting on the cable. Substituting Eq. 2 in Eq. 1:

$$N = (2T_{in} - f)sin\left(\frac{\phi}{2}\right) \tag{3}$$

From the Coulomb friction model, the friction force, f, can be expressed as:

$$f = sgn(V_{cable)}\mu N \tag{4}$$

where μ is the Coulomb friction coefficient and V_{cable} is the velocity of the cable relative to the sheath and considered positive if raising the arm (cable travels towards input), and negative if lowering the arm (cable travels towards output). Substituting Eq. 3 in Eq. 4 it is obtained:

$$f = sgn(V_{cable})\mu(2T_{in} - f)sin\left(\frac{\phi}{2}\right) \tag{5}$$

and solving for f:

$$f = sgn(V_{cable})\frac{2T_{in}\mu sin\left(\frac{\phi}{2}\right)}{1 + sgn(V_{cable})\mu sin\left(\frac{\phi}{2}\right)} \tag{6}$$

Considering inertial effects negligible, the output tension of a Bowden-sheath system can be computed as:

$$T_{out} = T_{in}\left(1 - sgn(V_{cable})\frac{2\mu sin\left(\frac{\phi}{2}\right)}{1 + sgn(V_{cable})\mu sin\left(\frac{\phi}{2}\right)}\right) \tag{7}$$

2 Gravity assistance strategy

The gravity torque acting on the shoulder was approximated as:

$$\tau_{sh} = m_a \cdot g \cdot l_{com} \cdot sin(\theta_{AOE}) \tag{8}$$

where m_a is the arm's total mass, g is the gravity acceleration vector (9.81 m/s^2), l_{com} is the distance of the arm center of mass from the shoulder center of rotation, and θ_{AOE} is the humeral angle of elevation estimated from the IMU. m_a and l_{com} were estimated from the user's height and weight using the anthropometric tables presented in Winter, 2009. The effect of humeral rotation and elbow flexion on shoulder torque was neglected and the model assumes a fully extended elbow.

The geometrical model shown in Fig. S2 was employed to compute the tendon tension necessary to guarantee the estimated assistive shoulder torque. The segment connecting the shoulder center of rotation and the anchor point on the upper arm can be defined as:

$$w_1 = \sqrt{d_{a1}^2 + r_{an}^2} \tag{9}$$

where d_{a1} is the distance between the upper arm anchor point projected onto the humerus line and the center of rotation of the shoulder, and r_{an} is the distance between the anchor point and the humerus axis. With the same quantities, the angle ϕ can be computed as:

$$\phi = tan^{-1}\left(\frac{r_{an}}{d_{a1}}\right) \tag{10}$$

By exploiting the Carnot theorem, the square of the shoulder cable length can be defined as:

$$l_{c1}^2 = (h + r_{ac})^2 + w_1^2 - 2 \cdot (h + r_{ac}) \cdot w \cdot cos(\pi - \theta_{AOE} - \phi) \tag{11}$$

where h is the height of the exit point of the cable above the skin on the shoulder, and r_{ac} is the acromion radius. With the Carnot theorem, one can also write:

$$(h + r_{ac})^2 = l_{c1}^2 + w_1^2 - 2 \cdot l_{c1} \cdot w_1 \cdot cos(\psi) \tag{12}$$

where ψ is the angle between the shoulder cable and w_1. Substituting (11) in (12), and solving for ψ, one obtains:

$$\psi = cos^{-1}\left(\frac{w_1 - (h + r_{ac}) \cdot cos(\pi - \theta_{AOE} - \phi)}{\sqrt{(h + r_{ac}^2) + w_1^2 - 2 \cdot (h + r_{ac}) \cdot w \cdot cos(\pi - \theta_{AOE} - \phi)}}\right) \tag{13}$$

Finally, the desired tendon tension for the shoulder support can be computed as:

$$T_{out,des} = \frac{\tau_{sh}}{w_1 \cdot sin(\psi)} \tag{14}$$

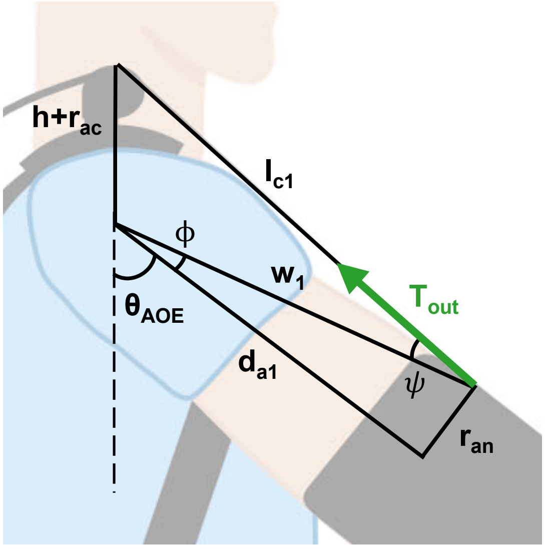

Figure S2: Shoulder torque transmission model. l_{c1} is the shoulder cable length, r_{ac} is the acromion radius, r_{an} is the distance between the anchor point and the humerus axis, d_{a1} is the distance of the anchor point from the center of rotation of the shoulder, h is the height of the exit point of the cable above the skin on the shoulder, θ_{AOE} is the shoulder angle of elevation, w_1, ϕ, and ψ are geometrical quantities useful to define the model, and T_{out} is the cable tension applied to support the shoulder.

3 Questionnaries

To evaluate discomfort participants filled out a 16-site modified Nordic Questionnaire with sites ranging from the upper neck to lower back and hands. Each site presented a 7-point Likert scale, ranging from ”No Discomfort” to ”Extreme Discomfort”. To evaluate perceived exertion, the Borg Rate of Perceived Exertion (RPE) scale was used. Perceived muscular fatigue was evaluated using a 6-site (pectoralis major, deltoids, trapezius, biceps, triceps, latissimus dorsi) diagram with a 7-point Likert scale ranging from ”No Fatigue” to ”Extreme Fatigue”.

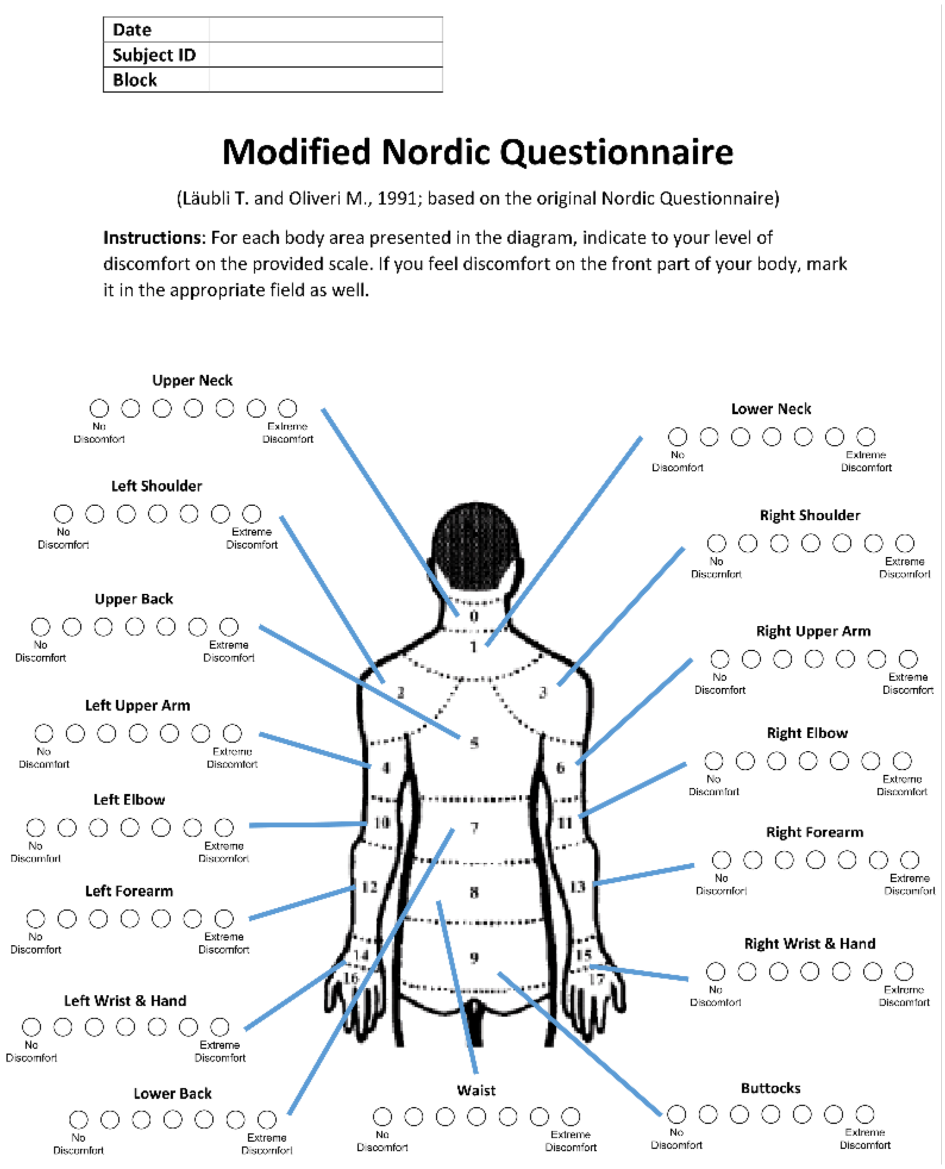

**Date**	
**Subject ID**	
**Block**	

Modified Nordic Questionnaire

(Läubli T. and Oliveri M., 1991; based on the original Nordic Questionnaire)

Instructions: For each body area presented in the diagram, indicate to your level of discomfort on the provided scale. If you feel discomfort on the front part of your body, mark it in the appropriate field as well.

**Date**	
**Subject ID**	
**Block**	

BORG Rate of Perceived Exertion

Instructions: How difficult did it feel performing the last activity? Please indicate your level of exertion by circling one of the scores on the table below.

**Borg RPE**	
**Score**	**Level of exertion**
6	No exertion at all
7	
7.5	Extremely light
8	
9	Very light
10	
11	Light
12	
13	Somewhat hard
14	
15	Hard (heavy)
16	
17	Very hard
18	
19	Extremely hard
20	Maximal exertion

**Date**	
**Subject ID**	
**Block**	

Muscle fatigue

Instructions: For each body area presented in the diagram related to the right side of the body, indicate to your level of muscle fatigue on the provided scale.

Pectoralis Major

○ ○ ○ ○ ○ ○ ○
No Fatigue — Extreme Fatigue

Deltoids

○ ○ ○ ○ ○ ○ ○
No Fatigue — Extreme Fatigue

Trapezius

○ ○ ○ ○ ○ ○ ○
No Fatigue — Extreme Fatigue

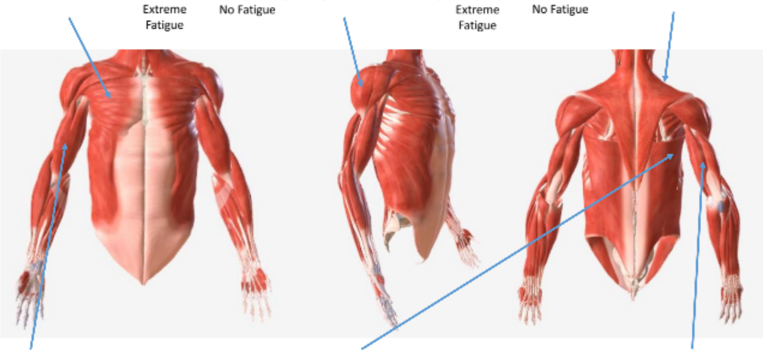

Biceps brachii

○ ○ ○ ○ ○ ○ ○
No Fatigue — Extreme Fatigue

Latissimus dorsi

○ ○ ○ ○ ○ ○ ○
No Fatigue — Extreme Fatigue

Triceps brachii

○ ○ ○ ○ ○ ○ ○
No Fatigue — Extreme Fatigue

4 Supplementary Data

4.1 Torque/tension tracking

	V1 ($v_{peak} = 60\frac{\circ}{s}$)			V2 ($v_{peak} = 120\frac{\circ}{s}$)			V3 ($v_{peak} = 180\frac{\circ}{s}$)		
**Pct. Err. RMSE (%)**	**Entire**	**Raise**	**Lower**	**Entire**	**Raise**	**Lower**	**Entire**	**Raise**	**Lower**
**Pre**	27.5	23.7	29.7	35.8	29.0	39.2	61.5	45.1	72.0
**25%**	24.2	22.8	24.5	30.5	28.2	31.4	38.3	33.6	41.6
**50%**	17.4	17.3	17.0	18.2	17.7	18.1	23.4	20.6	25.1

Table S1: RMSE of the percentage error presented for all combinations of velocities and support conditions, averaged across all participants and repetitions. These values are identical for both tension and torque, as the kinematic model relating the two quantities is linear. The results are further split showing the average RMSE of the entire arm cycle, the raising portion, and the lowering portion.

	V1 ($v_{peak} = 60\frac{\circ}{s}$)		V2 ($v_{peak} = 120\frac{\circ}{s}$)		V3 ($v_{peak} = 180\frac{\circ}{s}$)	
	**Torque Pct. Error (%)**	**Normalized Time (%)**	**Torque Pct. Error (%)**	**Normalized Time (%)**	**Torque Pct. Error (%)**	**Normalized Time (%)**
**Pre**	-29.0 (51.5)	20.4 (18.6)	-32.3 (66.3)	10.4 (10.8)	-3.20 (112)	17.4 (16.4)
**25%**	-32.3 (44.3)	17.2 (16.6)	-32.1 (64.5)	10.6 (10.7)	-37.5 (70.1)	12.0 (11.3)
**50%**	-30.0 (24.8)	18.2 (17.3)	-39.4 (16.9)	7.80 (8.63)	-43.7 (23.9)	8.27 (8.69)

Table S2: Peak percentage errors for the assistive torques during the raising phase, averaged across all participants and repetitions with standard deviation in brackets. Normalized time and standard deviation for the peak values are reported as well.

	V1 ($v_{peak} = 60\frac{\circ}{s}$)		V2 ($v_{peak} = 120\frac{\circ}{s}$)		V3 ($v_{peak} = 180\frac{\circ}{s}$)	
	**Torque Pct. Error (%)**	**Normalized Time (%)**	**Torque Pct. Error (%)**	**Normalized Time (%)**	**Torque Pct. Error (%)**	**Normalized Time (%)**
**Pre**	76.8 (54.1)	68.4 (14.2)	94.4 (71.0)	60.5 (7.76)	155 (90.3)	61.2 (5.48)
**25%**	58.0 (53.0)	74.0 (17.5)	80.0 (63.0)	61.4 (8.55)	99.2 (56.5)	62.3 (6.06)
**50%**	34.7 (29.9)	74.1 (17.3)	48.8 (25.3)	63.6 (8.95)	62.6 (29.2)	63.6 (6.75)

Table S3: Peak percentage errors for the assistive torque from the exosuit during lowering phase, averaged across all participants and repetitions with standard deviation in brackets. Normalized time and standard deviation for the peak values are reported as well.

4.2 Kinematics

outcome	speed	no	pre	25%	50%	p-value (C)	p-value (V)	p-value (C x V)
RMSE (°)	V1	4.755 (0.299)	4.389 (0.301)	4.514 (0.29)	4.874 (0.309)	0.335	< **0.001**	0.636
	V2	8.356 (0.584)	7.769 (0.396)	8.261 (0.462)	8.345 (0.446)			
	V3	12.524 (0.868)	13.586 (0.815)	13.586 (0.862)	13.888 (0.948)			
SPARC (-)	V1	2.76 (0.05)	2.752 (0.041)	2.789 (0.048)	2.832 (0.055)	**0.023**	< **0.001**	0.997
	V2	2.473 (0.046)	2.476 (0.044)	2.508 (0.047)	2.554 (0.053)			
	V3	2.349 (0.042)	2.368 (0.041)	2.377 (0.041)	2.416 (0.046)			

Table S4: Average shoulder position RMSE and SPARC, with standard deviation in brackets, across each velocity and condition are reported along with the p-values resulting from the GLMM considering condition, speed, and the interaction of condition and speed as fixed effects. The p-values for significant effects ($p < 0.05$) are shown in bold. SPARC scores are traditionally always negative, but were converted to positive values for the statistical tests.

	pre - no		25% - no		50% - no		25% - pre		50% - pre		50% - 25%	
outcome	change	p-value	change	p-value	change	p-value	change	p-value	change	p-value	change	p-value
RMSE (°)	N/A	N/A	N/A	N/A	N/A	N/A	N/A	N/A	N/A	N/A	N/A	N/A
SPARC (-)	0.005 (0.021)	0.809	0.031 (0.023)	0.552	0.073 (0.027)	**0.04**	0.025 (0.021)	0.552	0.068 (0.025)	**0.04**	0.042 (0.026)	0.42

Table S5: Pairwise comparisons across conditions for SPARC. Post-hoc pairwise comparisons for shoulder position RMSE were not performed since the effect of condition was not significant. The p-values for significant comparisons ($p < 0.05$) are shown in bold. A positive change in SPARC means that the first condition was less smooth than the second.

	V1-V2		V1-V3		V2-V3	
outcome	change	p-value	change	p-value	change	p-value
RMSE (°)	-3.55 (0.249)	< **0.001**	-8.756 (0.511)	< **0.001**	-5.207 (0.431)	< **0.001**
SPARC (-)	0.28 (0.022)	< **0.001**	0.406 (0.02)	< **0.001**	0.125 (0.021)	< **0.001**

Table S6: Pairwise comparisons across velocities for changes in RMSE and SPARC. The p-values for significant comparisons ($p < 0.05$) are shown in bold. A negative change in RMSE means that the first velocity had greater tracking errors than the second. A positive change in SPARC means that the first condition was less smooth than the second.

4.3 EMG

Outcome		no	pre	25%	50%	p-value (C)	p-value (V)	p-value (C x V)
Ant. Delt.	V1	0.435 (0.037)	0.358 (0.028)	0.324 (0.025)	0.285 (0.027)	$<$ **0.001**	0.116	0.996
	V2	0.459 (0.039)	0.380 (0.026)	0.353 (0.027)	0.307 (0.024)			
	V3	0.437 (0.037)	0.375 (0.027)	0.338 (0.027)	0.305 (0.027)			
Med. Delt.	V1	0.392 (0.043)	0.401 (0.037)	0.384 (0.036)	0.327 (0.031)	$<$ **0.001**	$<$ **0.001**	0.846
	V2	0.419 (0.045)	0.445 (0.042)	0.440 (0.042)	0.369 (0.036)			
	V3	0.434 (0.049)	0.493 (0.047)	0.457 (0.046)	0.427 (0.043)			
Post. Delt.	V1	0.415 (0.035)	0.447 (0.036)	0.439 (0.037)	0.480 (0.046)	**0.005**	**0.002**	0.837
	V2	0.454 (0.038)	0.482 (0.035)	0.492 (0.035)	0.507 (0.037)			
	V3	0.461 (0.039)	0.499 (0.035)	0.499 (0.039)	0.576 (0.046)			
Biceps	V1	0.485 (0.040)	0.619 (0.063)	0.573 (0.058)	0.519 (0.051)	**0.006**	0.754	0.754
	V2	0.438 (0.034)	0.478 (0.035)	0.492 (0.040)	0.446 (0.038)			
	V3	0.353 (0.029)	0.366 (0.028)	0.389 (0.031)	0.358 (0.039)			
Triceps	V1	0.471 (0.055)	0.445 (0.043)	0.434 (0.039)	0.426 (0.046)	0.061	0.87	0.87
	V2	0.492 (0.053)	0.487 (0.040)	0.475 (0.041)	0.425 (0.036)			
	V3	0.492 (0.057)	0.483 (0.043)	0.454 (0.041)	0.445 (0.039)			
Trapezius	V1	0.491 (0.046)	0.400 (0.032)	0.380 (0.032)	0.311 (0.029)	$<$ **0.001**	0.985	0.985
	V2	0.474 (0.042)	0.391 (0.029)	0.354 (0.027)	0.289 (0.024)			
	V3	0.422 (0.039)	0.325 (0.028)	0.307 (0.023)	0.254 (0.022)			
Lat. Dorsi	V1	0.395 (0.024)	0.387 (0.025)	0.386 (0.028)	0.360 (0.034)	0.838	0.838	0.838
	V2	0.424 (0.023)	0.428 (0.027)	0.437 (0.032)	0.402 (0.033)			
	V3	0.460 (0.028)	0.473 (0.034)	0.490 (0.036)	0.495 (0.043)			
Pectoralis	V1	0.548 (0.075)	0.431 (0.051)	0.401 (0.045)	0.347 (0.041)	$<$ **0.001**	0.999	0.999
	V2	0.511 (0.067)	0.398 (0.041)	0.376 (0.041)	0.334 (0.039)			
	V3	0.442 (0.056)	0.360 (0.041)	0.335 (0.043)	0.291 (0.035)			

Table S7: Average normalized iEMG for the entire arm cycle, with standard deviation in brackets, across each velocity (V) and condition (C). The p-values are given considering condition, speed, and the interaction of condition and speed as fixed effects. The p-values for significant effects ($p < 0.05$) are shown in bold.

Outcome		no	pre	25%	50%	p-value (C)	p-value (V)	p-value (C x V)
Ant. Delt.	V1	0.548 (0.045)	0.459 (0.035)	0.425 (0.033)	0.369 (0.038)	< **0.001**	< **0.001**	0.989
	V2	0.629 (0.053)	0.532 (0.035)	0.502 (0.038)	0.449 (0.035)			
	V3	0.622 (0.053)	0.530 (0.037)	0.479 (0.037)	0.452 (0.040)			
Med. Delt.	V1	0.486 (0.054)	0.494 (0.047)	0.477 (0.046)	0.401 (0.042)	< **0.001**	< **0.001**	0.859
	V2	0.583 (0.064)	0.611 (0.058)	0.614 (0.058)	0.524 (0.050)			
	V3	0.639 (0.076)	0.705 (0.067)	0.657 (0.066)	0.620 (0.062)			
Post. Delt.	V1	0.488 (0.045)	0.503 (0.043)	0.491 (0.043)	0.483 (0.041)	0.695	< **0.001**	0.928
	V2	0.580 (0.054)	0.611 (0.049)	0.616 (0.049)	0.602 (0.049)			
	V3	0.612 (0.059)	0.652 (0.050)	0.642 (0.054)	0.686 (0.061)			
Biceps	V1	0.537 (0.029)	0.686 (0.067)	0.647 (0.061)	0.569 (0.051)	**0.009**	< **0.001**	0.55
	V2	0.517 (0.024)	0.560 (0.032)	0.586 (0.042)	0.542 (0.048)			
	V3	0.432 (0.024)	0.427 (0.030)	0.465 (0.035)	0.433 (0.052)			
Triceps	V1	0.543 (0.069)	0.509 (0.056)	0.497 (0.051)	0.466 (0.056)	**0.01**	< **0.001**	0.974
	V2	0.610 (0.071)	0.616 (0.057)	0.605 (0.058)	0.531 (0.051)			
	V3	0.644 (0.079)	0.632 (0.060)	0.590 (0.058)	0.550 (0.054)			
Trapezius	V1	0.595 (0.049)	0.509 (0.038)	0.483 (0.038)	0.367 (0.030)	< **0.001**	**0.003**	0.953
	V2	0.620 (0.050)	0.551 (0.037)	0.492 (0.033)	0.395 (0.030)			
	V3	0.574 (0.047)	0.465 (0.036)	0.446 (0.029)	0.356 (0.028)			
Lat. Dorsi	V1	0.463 (0.026)	0.447 (0.028)	0.449 (0.032)	0.377 (0.030)	**0.003**	< **0.001**	0.993
	V2	0.537 (0.028)	0.525 (0.030)	0.538 (0.041)	0.458 (0.037)			
	V3	0.593 (0.036)	0.580 (0.038)	0.600 (0.042)	0.527 (0.042)			
Pectoralis	V1	0.598 (0.065)	0.485 (0.049)	0.463 (0.044)	0.377 (0.037)	< **0.001**	< **0.001**	0.995
	V2	0.595 (0.060)	0.474 (0.041)	0.443 (0.039)	0.379 (0.035)			
	V3	0.536 (0.054)	0.413 (0.039)	0.385 (0.043)	0.324 (0.032)			

Table S8: Average normalized iEMG for the raising phase, with standard deviation in brackets, across each velocity (V) and condition (C). The p-values are given considering condition, speed, and the interaction of condition and speed as fixed effects. The p-values for significant effects ($p < 0.05$) are shown in bold.

Outcome		no	pre	25%	50%	p-value (C)	p-value (V)	p-value (C x V)
Ant. Delt.	V1	0.320 (0.032)	0.259 (0.024)	0.226 (0.021)	0.200 (0.024)	< **0.001**	< **0.001**	0.992
	V2	0.294 (0.028)	0.241 (0.020)	0.214 (0.019)	0.174 (0.018)			
	V3	0.261 (0.026)	0.221 (0.019)	0.196 (0.019)	0.162 (0.018)			
Med. Delt.	V1	0.296 (0.034)	0.311 (0.030)	0.293 (0.028)	0.252 (0.025)	< **0.001**	**0.006**	0.896
	V2	0.264 (0.028)	0.292 (0.028)	0.279 (0.027)	0.225 (0.024)			
	V3	0.242 (0.026)	0.283 (0.028)	0.258 (0.027)	0.237 (0.027)			
Post. Delt.	V1	0.341 (0.033)	0.388 (0.036)	0.381 (0.035)	0.455 (0.057)	< **0.001**	0.171	0.788
	V2	0.330 (0.030)	0.357 (0.029)	0.369 (0.030)	0.403 (0.037)			
	V3	0.318 (0.031)	0.344 (0.029)	0.352 (0.033)	0.447 (0.048)			
Biceps	V1	0.432 (0.054)	0.555 (0.070)	0.507 (0.064)	0.472 (0.062)	**0.006**	< **0.001**	0.934
	V2	0.357 (0.042)	0.399 (0.041)	0.402 (0.043)	0.359 (0.038)			
	V3	0.275 (0.032)	0.302 (0.031)	0.310 (0.033)	0.279 (0.032)			
Triceps	V1	0.399 (0.050)	0.380 (0.038)	0.370 (0.035)	0.380 (0.045)	0.325	**0.006**	0.683
	V2	0.374 (0.044)	0.361 (0.032)	0.348 (0.032)	0.316 (0.030)			
	V3	0.345 (0.047)	0.327 (0.032)	0.312 (0.030)	0.325 (0.033)			
Trapezius	V1	0.392 (0.055)	0.291 (0.034)	0.275 (0.032)	0.256 (0.041)	< **0.001**	< **0.001**	0.896
	V2	0.334 (0.049)	0.236 (0.026)	0.218 (0.025)	0.185 (0.024)			
	V3	0.273 (0.039)	0.179 (0.024)	0.160 (0.019)	0.148 (0.019)			
Lat. Dorsi	V1	0.327 (0.031)	0.325 (0.026)	0.320 (0.028)	0.336 (0.041)	0.085	**0.003**	0.515
	V2	0.314 (0.025)	0.337 (0.029)	0.341 (0.030)	0.343 (0.035)			
	V3	0.334 (0.028)	0.368 (0.039)	0.379 (0.037)	0.452 (0.053)			
Pectoralis	V1	0.504 (0.092)	0.373 (0.056)	0.340 (0.050)	0.308 (0.047)	< **0.001**	< **0.001**	0.973
	V2	0.431 (0.076)	0.319 (0.045)	0.302 (0.044)	0.281 (0.044)			
	V3	0.355 (0.059)	0.297 (0.045)	0.277 (0.044)	0.248 (0.040)			

Table S9: Average normalized iEMG for the lowering phase, with standard deviation in brackets, across each velocity (V) and condition (C). The p-values are given considering condition, speed, and the interaction of condition and speed as fixed effects. The p-values for significant effects ($p < 0.05$) are shown in bold.

Whole	pre - no			25% - no			50% - no			25% - pre			50% - pre			50% - 25%		
Muscle	change		p-value	change		p-value	change		p-value	change		p-value	change		p-value	change		p-value
Ant. delt.	-0.072	(-16.3%)	< **0.001**	-0.105	(-23.8%)	< **0.001**	-0.145	(-32.6%)	< **0.001**	-0.033	(-9.0%)	**0.003**	-0.073	(-19.5%)	< **0.001**	-0.039	(-11.6%)	**0.003**
Med. delt.	0.030	(7.3%)	0.359	0.011	(2.7%)	0.566	-0.042	(-10.2%)	0.131	-0.019	(-4.2%)	0.398	-0.072	(-16.3%)	< **0.001**	-0.054	(-12.6%)	**0.003**
Post. delt.	0.032	(7.3%)	0.184	0.033	(7.4%)	0.184	0.076	(17.2%)	**0.003**	0.001	(0.2%)	0.963	0.044	(9.2%)	0.110	0.043	(9.1%)	0.136
Biceps	0.055	(13.1%)	**0.005**	0.057	(13.6%)	**0.006**	0.014	(3.3%)	0.529	0.002	(0.5%)	0.924	-0.041	(-8.6%)	0.093	-0.043	(-9.1%)	0.087
Triceps	-0.013	(-2.8%)	0.675	-0.030	(-6.3%)	0.675	-0.053	(-10.9%)	0.224	-0.017	(-3.6%)	0.675	-0.040	(-8.4%)	0.107	-0.023	(-5.0%)	0.650
Lat. dorsi	0.002	(0.5%)	1.000	0.010	(2.2%)	1.000	-0.010	(-2.4%)	1.000	0.008	(1.8%)	1.000	-0.012	(-2.9%)	1.000	-0.020	(-4.6%)	1.000
Pectoralis	-0.103	(-20.6%)	**0.001**	-0.128	(-25.8%)	< **0.001**	-0.175	(-35.1%)	< **0.001**	-0.026	(-6.5%)	0.104	-0.072	(-18.2%)	< **0.001**	-0.046	(-12.6%)	**0.013**
Trapezius	-0.090	(-19.6%)	< **0.001**	-0.116	(-25.1%)	< **0.001**	-0.178	(-38.5%)	< **0.001**	-0.025	(-6.8%)	**0.041**	-0.087	(-23.5%)	< **0.001**	-0.062	(-17.9%)	< **0.001**

Table S10: Pairwise comparisons across conditions for changes in iEMG for the entire movement cycle. The p-values for significant comparisons ($p < 0.05$) are shown in bold. A negative change in iEMG means that the muscle activation was higher in the second condition.

Raising	pre - no			25% - no			50% - no			25% - pre			50% - pre			50% - 25%		
Muscle	change		p-value	change		p-value	change		p-value	change		p-value	change		p-value	change		p-value
Ant. delt.	-0.093	(-15.5%)	< **0.001**	-0.131	(-21.9%)	< **0.001**	-0.177	(-29.6%)	< **0.001**	-0.038	(-7.6%)	**0.019**	-0.084	(-16.7%)	< **0.001**	-0.046	(-9.8%)	**0.019**
Med. delt.	0.031	(5.5%)	0.776	0.012	(2.1%)	0.776	-0.059	(-10.4%)	0.168	-0.020	(-3.3%)	0.776	-0.090	(-15.1%)	< **0.001**	-0.071	(-12.2%)	**0.010**
Post. delt.	0.028	(4.9%)	1.000	0.021	(3.8%)	1.000	0.027	(4.8%)	1.000	-0.006	(-1.1%)	1.000	-0.001	(-0.1%)	1.000	0.006	(1.0%)	1.000
Biceps	0.055	(11.1%)	**0.019**	0.068	(13.8%)	**0.007**	0.018	(3.7%)	0.529	0.013	(2.4%)	0.666	-0.036	(-6.7%)	0.287	-0.050	(-8.9%)	0.162
Triceps	-0.014	(-2.3%)	0.806	-0.035	(-5.9%)	0.806	-0.083	(-13.9%)	0.074	-0.021	(-3.7%)	0.806	-0.069	(-11.8%)	**0.016**	-0.047	(-8.5%)	0.120
Lat. dorsi	-0.014	(-2.7%)	1.000	-0.003	(-0.6%)	1.000	-0.078	(-14.8%)	**0.001**	0.011	(2.2%)	1.000	-0.064	(-12.5%)	**0.014**	-0.075	(-14.3%)	**0.013**
Pectoralis	-0.119	(-20.7%)	< **0.001**	-0.146	(-25.4%)	**0.000**	-0.216	(-37.6%)	< **0.001**	-0.027	(-5.9%)	0.150	-0.097	(-21.3%)	< **0.001**	-0.070	(-16.3%)	< **0.001**
Trapezius	-0.089	(-14.9%)	**0.001**	-0.123	(-20.6%)	**0.000**	-0.223	(-37.5%)	< **0.001**	-0.034	(-6.7%)	**0.044**	-0.135	(-26.6%)	< **0.001**	-0.101	(-21.3%)	< **0.001**

Table S11: Pairwise comparisons across conditions for changes in iEMG for the raising phase. The p-values for significant comparisons ($p < 0.05$) are shown in bold. A negative change in iEMG means that the muscle activation was higher in the second condition.

Lowering	pre - no			25% - no			50% - no			25% - pre			50% - pre			50% - 25%		
Muscle	change		p-value	change		p-value	change		p-value	change		p-value	change		p-value	change		p-value
Ant. delt.	-0.051	(-17.6%)	< **0.001**	-0.079	(-27.2%)	< **0.001**	-0.113	(-38.9%)	< **0.001**	-0.028	(-11.6%)	**0.001**	-0.062	(-25.8%)	< **0.001**	-0.034	(-16.0%)	**0.001**
Med. delt.	0.029	(10.9%)	0.079	0.010	(3.7%)	0.432	-0.029	(-10.8%)	0.091	-0.019	(-6.5%)	0.121	-0.058	(-19.6%)	< **0.001**	-0.039	(-14.0%)	**0.005**
Post. delt.	0.032	(9.8%)	**0.027**	0.038	(11.4%)	**0.024**	0.105	(31.7%)	< **0.001**	0.005	(1.4%)	0.667	0.072	(19.9%)	**0.004**	0.067	(18.3%)	**0.009**
Biceps	0.057	(16.3%)	**0.004**	0.049	(14.0%)	**0.013**	0.012	(3.5%)	0.525	-0.008	(-1.9%)	0.667	-0.044	(-10.9%)	**0.020**	-0.037	(-9.2%)	0.061
Triceps	-0.016	(-4.4%)	0.974	-0.029	(-7.9%)	0.974	-0.033	(-8.8%)	0.974	-0.013	(-3.7%)	0.974	-0.016	(-4.6%)	0.974	-0.003	(-1.0%)	0.974
Lat. dorsi	0.018	(5.4%)	0.667	0.021	(6.5%)	0.643	0.048	(14.8%)	0.137	0.003	(1.0%)	0.828	0.030	(8.9%)	0.667	0.027	(7.8%)	0.667
Pectoralis	-0.098	(-23.0%)	**0.008**	-0.120	(-28.3%)	**0.002**	-0.148	(-34.8%)	< **0.001**	-0.023	(-6.9%)	0.179	-0.051	(-15.4%)	**0.010**	-0.028	(-9.1%)	0.179
Trapezius	-0.099	(-30.0%)	< **0.001**	-0.117	(-35.5%)	< **0.001**	-0.138	(-42.0%)	< **0.001**	-0.018	(-7.9%)	**0.140**	-0.040	(-17.1%)	**0.011**	-0.021	(-10.0%)	0.140

Table S12: Pairwise comparisons across conditions for changes in iEMG for the lowering phase. The p-values for significant comparisons ($p < 0.05$) are shown in bold. A negative change in iEMG means that the muscle activation was higher in the second condition.

Muscle group	pre-no	25%-no	50%-no	25%-pre	50%-pre	50%-25%
Ant. delt.	**	**	**	*	**	*
Med. delt.					**	*
Post. delt.			*			
Biceps	*	*				
Triceps						
Trapezius	**	**	**	*	**	**
Lat. dorsi						
Pectoralis major	**	**	**		**	*

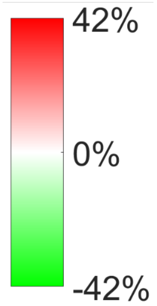

Table S13: EMG statistical results for the pairwise post-hoc tests comparing changes in muscular activity across all muscle groups for the entire arm cycle. The following significance codes are used for the p-values: ** = [0, 0.001), * = [0.001, 0.05), and a blank space represents [0.05, 1]. The percentage changes in normalized iEMG, relative to the first condition being compared, is coded in color, with green representing a decrease in iEMG from the second to the first condition, and red representing an increase.

References

Winter, D. (2009). Anthropometry. In *Biomechanics and motor control of human movement* (pp. 82–106). John Wiley & Sons, Ltd. https://doi.org/https://doi.org/10.1002/9780470549148.ch4
